# Comparison of Electromechanical Delay during Ventricular Tachycardia and Fibrillation under Different Conductivity Conditions Using Computational Modeling

**DOI:** 10.1155/2020/9501985

**Published:** 2020-03-29

**Authors:** Aulia K. Heikhmakhtiar, Ki M. Lim

**Affiliations:** ^1^School of Computing, Telkom University, Bandung 40257, Indonesia; ^2^Department of IT Convergence Engineering, Kumoh National Institute of Technology, Gumi 39177, Republic of Korea

## Abstract

Electromechanical delay (EMD) is the time interval between local myocyte depolarization and the onset of myofiber shortening. Previously, researchers measured EMD during sinus rhythm and ectopic pacing in normal and heart failure conditions. However, to our knowledge, there are no reports regarding EMD during another type of rhythms or arrhythmia. The goal of this study was to quantify EMD during sinus rhythm, tachycardia, and ventricular fibrillation conditions. We hypothesized that EMD under sinus rhythm is longer due to isovolumetric contraction which is imprecise during arrhythmia. We used a realistic model of 3D electromechanical ventricles. During sinus rhythm, EMD was measured in the last cycle of cardiac systole under steady conditions. EMD under tachycardia and fibrillation conditions was measured during the entire simulation, resulting in multiple EMD values. We assessed EMD for the following 3 conduction velocities (CVs): 31 cm/s, 51 cm/s, and 69 cm/s. The average EMD during fibrillation condition was the shortest corresponding to 53.45 ms, 55.07 ms, and 50.77 ms, for the CVs of 31 cm/s, 51 cm/s, and 69 cm/s, respectively. The average EMD during tachycardia was 58.61 ms, 58.33 ms, and 52.50 ms for the three CVs. Under sinus rhythm with action potential duration restitution (APDR) slope 0.7, the average EMD was 66.35 ms, 66.41 ms, and 66.60 ms in line with the three CVs. This result supports our hypothesis that EMD under sinus rhythm is longer than that under tachyarrhythmia conditions. In conclusion, this study observed and quantified EMD under tachycardia and ventricular fibrillation conditions. This simulation study has widened our understanding of EMD in 3D ventricles under chaotic conditions.

## 1. Introduction

The heart is a vital organ that distributes oxygenated blood throughout the body. Any alteration in the electrical activation sequence in the heart diminishes the efficacy of its contractility. This can lead to abnormalities in perfusion, impaired pumping function, and, in chronic cases, asymmetric ventricular hypertrophy [1, 2]. The time interval of excitation-contraction in myocytes is approximately ten milliseconds. The term “excitation-contraction time interval” is defined as an electromechanical delay (EMD). The excitation time of myocytes is described as the local depolarization time (electrical activation time (EAT)), and the contraction time is known as the onset myofilament shortening (mechanical activation time (MAT)) [3, 4].

EMD is composed of two components: (a) intrinsic latent period between the depolarization and myofilament activation in the myocytes [5] and (b) local myofiber mechanical loading conditions in an intact heart [6]. Experimental observation has shown that EMD is nonuniform and eminently depends on the electrical activation sequence [1, 7–10]. In 2004, Ashikaga et al. showed that EMD at a specific location during sinus rhythm (SR) was different from that during epicardial pacing [8]. In 2007, Ashikaga et al. demonstrated that during transmural electrical propagation, EMD was longer in the late-activated region than at the corresponding stimuli site [7]. An experimental study by Russell et al. showed that EMD in the ventricle with the left bundle branch block (LBBB) was longer at the late-activated left ventricle (LV) free-wall than at the early septum [6]. Russell et al. also stated that mechanical loading condition also plays a role in determining EMD. These experimental studies on EMD measurements were limited to surface or transmural measurement. Measuring EMD in 3D ventricles is limited due to insufficient experimental tools, which are to observe electrical activation, mechanical activation, and local mechanical loading condition, simultaneously. Hence, researchers have managed to use realistic computational modeling to observe EMD distribution in 3D ventricles.

The first computational study that determined EMD using a 3D electromechanical model of the ventricle was conducted by Usyk and McCulloch [4]. They measured EMD distribution in 3D ventricles under SR and ectopic pacing conditions. Interestingly, they observed that EMD can be both positive and negative, meaning that myofiber contraction can occur before electrical activation of the 3D ventricle. The negatively activated region commonly found in the septal area can be described as unloading of the septum. Their findings aligned with the experimental findings of Wyman et al. [11]. In 2010, Gurev et al. used a similar approach to that used by Usyk and McCulloch, involving an electromechanical model of 3D ventricles to measure EMD [3]. They demonstrated that EMD is nonuniform under SR and ectopic pacing, depending on the electrical activation sequence, and this is consistent with the finding of the experimental study conducted by Ashikaga et al. in 2004. Gurev et al. also found that prestretched myocytes prolonged the onset of myofiber shortening resulting in prolonged EMD and contributed to heterogeneous 3D EMD distribution. In 2012, the computational study by Constantino et al. [12] showed that EMD increased in heart failure (HF), especially at the late-activated LV free-wall during LBBB (following the experimental study of Russell et al. in 2011). Furthermore, they showed that pacing in the longest EMD region increased hemodynamic performance using cardiac resynchronization therapy (CRT). In 2013, Constantino et al. [13] extended their work by systematically varying the remodeling factor of HF; the remodeled fiber/sheet architecture decreased electrical conduction, deranged Ca^2+^ handling, and reduced stiffness of the failing myocardium. They found that deranged Ca^2+^ significantly prolonged EMD in dyssynchronous HF. To observe the relationship between mechanical loading condition and EMD, our group computationally determined the influence of left ventricular assist device (LVAD) on EMD under four HF conditions from mild to severe HF [14]. Consistent with the experimental data, EMD was decreased by mechanical unloading with LVAD. In 2018, our group also performed a computational study on EMD in LBBB patients with the implementation of CRT and LVAD simultaneously [15]. The results showed that CRT and LVAD jointly reduced EMD significantly by restoring the electrical activation sequence and mechanical unloading, respectively, compared to CRT only in the LBBB heart.

To the best of our knowledge, no study has measured EMD under tachycardia or even ventricular fibrillation condition. This cannot be obtained through experimental methods; thus, we performed a computational study. The goal of this computational study was to compare EMD under SR with tachycardia and ventricular fibrillation conditions. We hypothesized that EMD under SR is longer due to mechanical loading, especially on isovolumetric contraction. SR simulation was divided into two groups: slope 0.7 and slope 1.8. We used two different slopes for the action potential duration restitution (APDR) curve to obtain different characteristics of electrical physiology. Previous studies showed that when the slope of the APDR curve is steeper than 1, it would lead to instability or alternans [16–18]. The alternans are prone to create a fragmented spiral wave, which represents a fibrillation condition [19–22]. To simulate tachycardia and fibrillation conditions, we used S1-S2 protocols for both cases. We assigned the slopes of the APDR curve 0.7 to tachycardia and 1.8 to fibrillation condition. We varied the conduction velocity (CV) for SR and chaotic conditions with three different categories including 31 cm/s, 51 cm/s, and 69 cm/s. We aimed to improve our understanding of the EMD phenomenon using an intact ventricle to provide possibilities for a novel treatment.

## 2. Materials and Methods

The electromechanical model of the 3D ventricle used in this study consisted of an electrophysiological model and mechanical or myofilament dynamics model, which were coupled via calcium transient following Gurev et al.'s study [3]. The electrophysiological model used in this study is in accordance with that used by Tusscher and Panfilov [23]. The mechanical model was in accordance with Rice et al. myofilament dynamic model [24]. The myofilament model mimicked the cross-bridge activation of actin and myosin. We used human ventricles geometry obtained from diffusion-tensor magnetic resonance imaging [25].

### 2.1. Simulation Protocol

We simulated three electrophysiological conditions of the human heart including SR, tachycardia, and ventricular fibrillation. We simulated three samples for each case with three different CVs: 31 cm/s, 51 cm/s, and 69 cm/s. All the simulations were conducted for 7 sec for both electrophysiological and mechanical contractions. In SR, we simulated two groups of the three CVs that had different slopes of the APDR curve: 0.7 and 1.8. APDR slope is known to be prone to alternans if the slope is >1. To adjust the slope of the APDR curve to be steeper than 1, we followed the parameter set used by Tusscher and Panfilov [18]. SR simulations were performed for 7 sec under an optimal initial state to reach a steady state with a cycle length of 600 ms. To simulate tachycardia and fibrillation conditions, we employed S1-S2 protocols, following previous studies [26]. S1 was applied three times at the apex as ectopic pacing, generating electrical waves toward the base of the ventricle. Then, we applied the S2 protocol as an artificial procedure, which sets half of the ventricle to the resting state. S2 was applied right after the third S1, while the electrical wave propagated halfway toward the base. The resting state of half of the ventricle induced the unfinished wavelength to generate the reentry conditions. The APDR slopes were 0.7 and 1.8 for reentry and VF conditions, respectively, for the three CVs. Therefore, we conducted simulation for twelve unique cases.

The mechanical simulation was run for the same duration (7 sec) in alignment with the electrical simulation. The mechanical simulator was reading calcium transient data which were obtained from the electrophysiological simulation. The calcium transient data were first transformed with the Gaussian point before it was read by the mechanical simulator. The contraction of the myofibril followed the electrical activation sequence. During tachycardia and fibrillation conditions, the ventricles were quivering instead of pumping normally. Since the ventricular mechanics were coupled with the lumped parameter model of the circulatory systems, we obtained LV and systemic artery (SA) pressures. To see a more detailed explanation for this electromechanical coupling technique, please refer to the study [27, 28].

**Figure 1:**
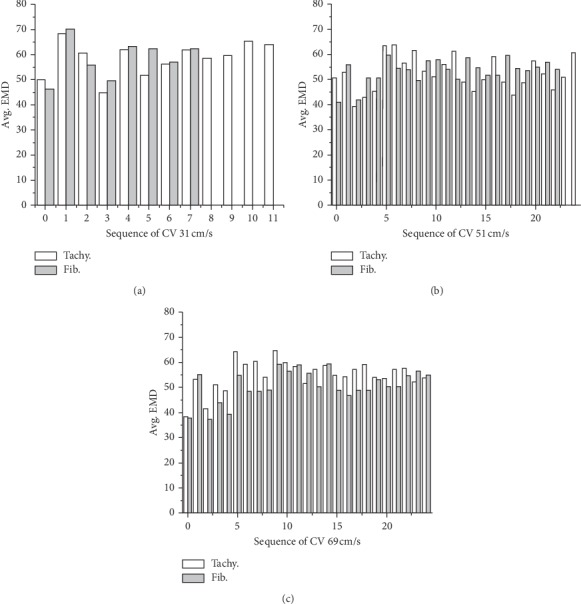
Multiple average electromechanical delay (EMD) comparison in tachycardia and fibrillation conditions for the conduction velocity of 31 cm/s, 51 cm/s, and 69 cm/s.

EMD was calculated from the time interval between local depolarization (when the membrane potential of the nodes exceeded −30 mV (EAT)) and the maximum myofiber stress (MAT). During SR, EMD was obtained from a cycle length that was already in a steady state for slopes 0.7 and 1.8. Under tachycardia and fibrillation conditions, EMD was measured during the whole simulation, including multiple EAT and MAT calculations, thus resulting in a series of EMD. Next, we calculated the average of EMD from each series and expressed it in Figure 1 according to the CVs. For the last comparison, we determined the average EMD based on the cases, which are SR slope 0.7, SR slope 1.8, tachycardia, and fibrillation conditions, categorized them based on the CVs, and presented them in Table 1 for comparison.

## 3. Results

**Figure 2:**
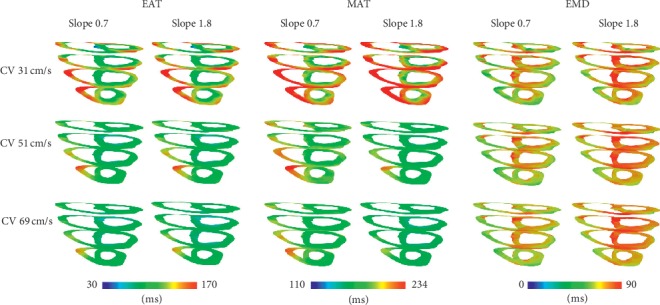
Electrical activation time (EAT), mechanical activation time (MAT), and electromechanical delay (EMD) comparison between sinus rhythm conditions with slopes of 0.7 and 1.8 under the conduction velocity of 31 cm/s, 51 cm/s, and 69 cm/s.

Figure 2 shows EAT, MAT, and EMD comparison between SRs with slopes of APDR curve 0.7 and 1.8 under three different CVs. Generally, EAT and MAT were shortened with an increase in CV, and EMD was longer for the slope of 1.8 than 0.7. The longest EMDs in slope 0.7 were 66.35 ms, 66.41 ms, and 66.60 ms under the CVs of 31 cm/s, 51 cm/s, and 69 cm/s, respectively. However, the longest EMDs in slope 1.8 were 72.25 ms, 72.78 ms, and 72.90 ms under the CVs of 31 cm/s, 51 cm/s, and 69 cm/s, respectively (see Table 1).

**Figure 3:**
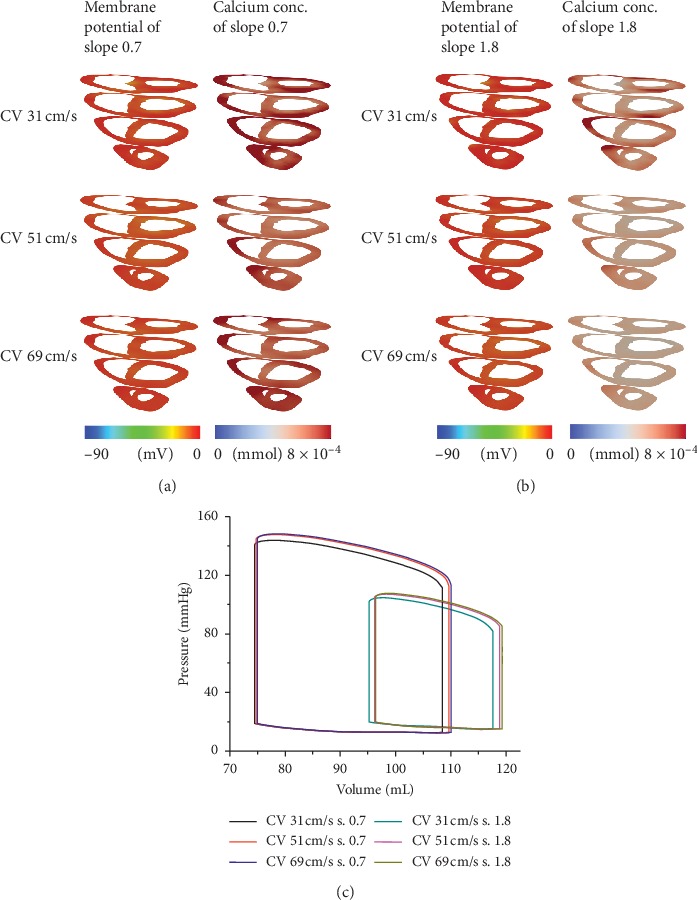
Membrane potential and calcium distribution of sinus rhythm (SR) with slope 0.7 (a) and slope 1.8 (b) and left ventricle (LV) pressure-volume loop under SR condition with slopes 0.7 and 1.8 (c) of the three conduction velocities.

Figure 3 shows the membrane potential and calcium distribution of SR with slopes of 0.7 and 1.8 with CV escalation during the depolarization of the whole ventricles (Figures 3(a) and 3(b)) and LV pressure-volume loop for all cases (Figure 3(c)). Calcium activation in slope 0.7 was higher than that in slope 1.8 under the three CV categories. The calcium directly affected the efficacy of ventricular pumping. As shown in Figure 3(c), higher calcium activation (in slope 0.7 cases) exhibited a wider area of pressure-volume relation than in slope 1.8 cases, with a slight difference in each CV. The stroke volume of SR with a slope of 0.7 was approximately 32 to 35 mL, and for a slope of 1.8, it was 23 to 25 mL. In addition, the maximum LV pressure range was between 144 to 149 mmHg and 104 to 108 mmHg, for slopes 0.7 and 1.8, respectively.

**Figure 4:**
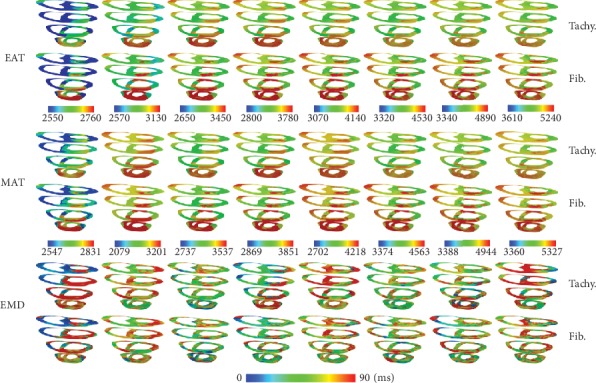
Electrical activation time (EAT), mechanical activation time (MAT), and electromechanical delay (EMD) comparison (in ms) between tachycardia and ventricular fibrillation conditions for the conduction velocity of 31 cm/s.

**Figure 5:**
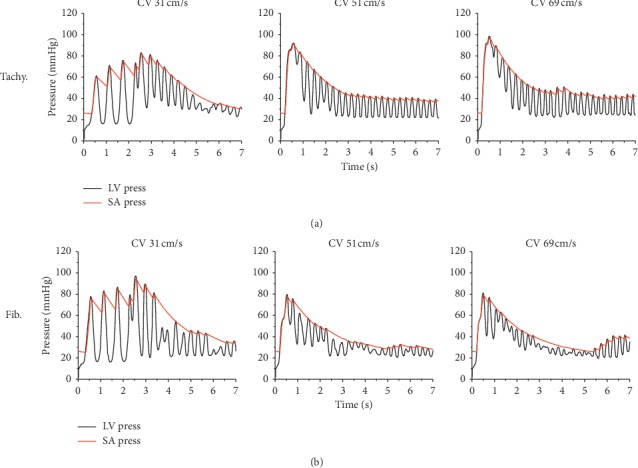
Left ventricle and systemic artery pressures in tachycardia (a) and ventricular fibrillation conditions (b) for the conduction velocity (CV) of 31 cm/s, 51 cm/s, and 69 cm/s.

Figure 4 shows EAT, MAT, and EMD under tachycardia and fibrillation conditions for the CV of 31 cm/s. EAT and MAT under the fibrillation condition were longer than those under the tachycardia condition. EAT was measured starting from 2550 ms to avoid S1 stimuli (3 times with cycle 600 ms) for the CV of 31 cm/s. The three times of S1 stimuli can be seen in Figure 5 on the left column as normal LV and SA pressure waveform three times before the chaotic condition. We obtained 12 series of EAT, MAT, and EMD from tachycardia measurements and 8 series for the fibrillation condition measurements. We only showed 8 series of 3D sliced ventricles to compare the distributions of EAT, MAT, and EMD under tachycardia and fibrillation conditions. The average EMD of each series during tachycardia and fibrillation is shown in Figure 1(a). The overall average EMD is also shown in Table 1 for comparison.

**Figure 6:**
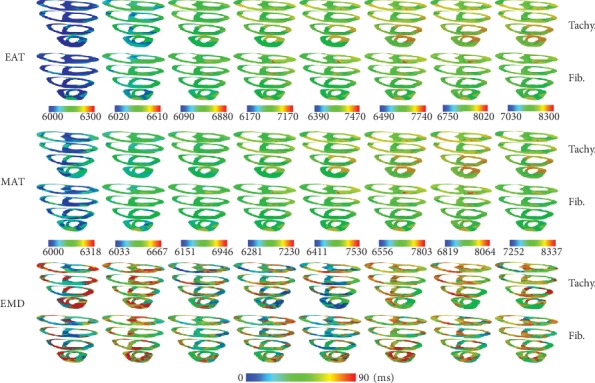
Electrical activation time (EAT), mechanical activation time (MAT), and electromechanical delay (EMD) comparison (in ms) between tachycardia and fibrillation conditions for the conduction velocity of 61 cm/s.

Figure 6 shows EAT, MAT, and EMD under tachycardia and fibrillation conditions for the CV of 51 cm/s. For the CV of 51 cm/s, EAT and MAT under the fibrillation condition were shorter than those under the tachycardia condition. The overall EAT and MAT for the CV of 51 cm/s were shorter than those for the CV of 31 cm/s. EAT for the CV of 51 cm/s was measured starting from 6000 ms because we did not apply the S1-S2 protocols for the CVs of 51 cm/s and 69 cm/s. Instead, the simulation started from the last period of the simulation, with the CV of 31 cm/s. However, we set the parameters that can be used to obtain the appropriate CVs. For EMD under tachycardia and fibrillation conditions, we obtained 25 and 24 series from the measurement, respectively. The average EMD for each series in tachycardia and fibrillation is displayed in Figure 1(b). Table 1 shows the overall average of EMD during tachycardia and fibrillation conditions.

**Figure 7:**
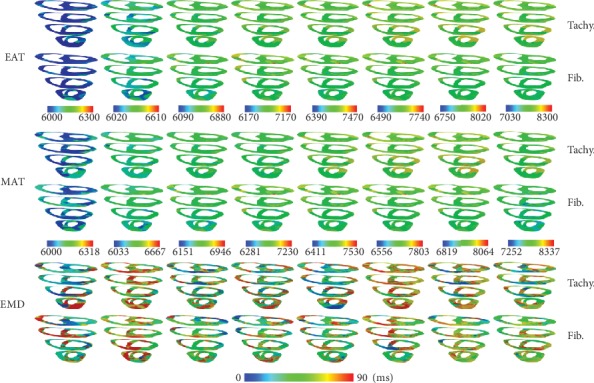
Electrical activation time (EAT), mechanical activation time (MAT), and electromechanical delay (EMD) comparison (in ms) between tachycardia and fibrillation conditions for the conduction velocity of 69 cm/s.

Figure 7 shows EAT, MAT, and EMD under tachycardia and fibrillation conditions for the CV of 69 cm/s. EAT and MAT in fibrillation condition were shorter than those in tachycardia condition for the CV of 69 cm/s. We obtained 25 series of EAT, MAT, and EMD for both tachycardia and fibrillation conditions from the measurement. EAT and MAT of CV 69 cm/s was the shortest among the three CVs. The average EMD in tachycardia and fibrillation conditions were shorter than that in SR conditions. The quantification of EMD is shown in Figure 1(b) and Table 1 during tachycardia and fibrillation conditions for the CV of 69 cm/s.

Figure 1 shows the average EMD comparison between tachycardia and fibrillation conditions for three different CVs. In Figure 1(a) (CV of 31 cm/s), the average EMD for tachycardia has twelve times activation, and there is eight times activation for the fibrillation condition obtained from the measurement. On average, EMDs for tachycardia and fibrillation were 58.61 ms and 53.45 ms, respectively, for the CV of 31 cm/s (Figure 1(a)). For the CV of 51 cm/s, the average EMDs for tachycardia and fibrillation were 58.33 and 55.07 ms, respectively (Figure 1(b)). The average EMDs for the CV of 69 cm/s during tachycardia and fibrillation were 52.50 and 50.77 ms, respectively (Figure 1(c); see Table 1).

Figure 5 shows the comparison of LV and SA pressures between tachycardia and fibrillation conditions for three different CVs. For the CV of 31 cm/s, the first three oscillations were the ventricular pumping from three times S1 stimuli in both tachycardia and fibrillation conditions. The chaotic conditions started at approximately 2.5 seconds. For the CVs of 51 cm/s and 69 cm/s, their simulation was not initiated by S1-S2 protocols; instead, their simulation was started by the last electrical signal state from that in the CV 31 cm/s. Hence, no regulated oscillation appeared during the first 3 sec of the simulation for the CVs of 51 cm/s and 69 cm/s. Overall, the LV and SA in tachycardia condition showed steady activation compared to those in fibrillation conditions. The average LV peak-pressure of tachycardia was higher than that in fibrillation condition for the CVs of 51 cm/s and 69 cm/s.

Table 1 shows the average EMD in all cases for three different CVs. These data support our hypothesis that EMD under SR is longer than that under tachycardia and fibrillation conditions. Under SR, the EMD of slope 0.7 is shorter than that of slope 1.8. However, in chaotic conditions, EMD in fibrillation with a 1.8 parameter setting was shorter than that under tachycardia condition with a 0.7 parameter setting.

## 4. Discussion

This work demonstrated the benefit of using computational simulation methods to expand our understanding of EMD behavior not only under SR conditions but also under tachycardia and fibrillation conditions. We utilized a realistic electromechanical model of ventricles representing contraction-excitation events in 3D ventricles to measure EMD. Excluding sinusal rhythm, we believe that this is the first work to investigate EMD during chaotic conditions including tachycardia and fibrillation conditions.

In general, the findings of this study are as follows:

1. The parameter setting between slopes 0.7 and 1.8 showed different calcium concentration activation during SR depolarization (Figures 3(a) and 3(b)). Calcium is the main factor for cross-bridge activation. As demonstrated in our previous work, the reduction in calcium level diminished fiber stress, thus prolonging EMD [14]. The lesser calcium level shown in SR with the slope of 1.8 not only exhibited longer EMD but also diminished systolic function. This was identified with lower LV pressure and volume (Figure 3(c)).
2. EMD in SR with the slope of 1.8 was longer than that with a slope of 0.7. However, under chaotic conditions, EMD in tachycardia (slope 0.7) was longer than that in fibrillation conditions (slope 1.8). It can be described that during tachycardia, electrical propagation with mechanical pumping occurs fast but steadily (Figure 5(a)). However, in fibrillation condition, contraction of the heart is more chaotic and highly uncoordinated (Figure 5(b)).
3. The difference in average EMD for 2 chaotic conditions (tachycardia and fibrillation conditions) was not significant when CV increased.

EMD is composed of two factors, which are the intrinsic latent period between depolarization and myofilament activation in the myocytes, and local myofiber mechanical loading conditions. The EMD calculation in this study was based on the time interval between the transmembrane voltage exceeding -30 mV and maximum myofiber stress. In a study by Gurev et al., the time interval was between depolarization passing 0 mV and 10% shortening of the myofiber consistent with that of Ashikaga et al. and Sengupta et al. [8, 10]. However, in another study by Usyk and McCulloch, EMD was defined as the time interval between the transmembrane at −40 mV and time of peak positive fiber strain [4]. The variation in defining MAT gives us more understanding of cardiac EMD.

In SR, the heart contracts in synchrony during systole giving some appropriate pressure to eject blood through the aortic valve. Before LV pressure exceeds aortic pressure, the isovolumetric contraction will occur for a few milliseconds. The isovolumetric contraction depends on myofiber stress and mechanical loading condition. Higher mechanical load and/or the lesser myofiber stress cause the longer EMD [6, 14]. However, in chaotic conditions such as tachycardia and fibrillation conditions, the heart shows uncoordinated contraction with ineffective systolic function [29–31]. Hence, our hypothesis is supported by this work.

In the normal heart, CV during electrical propagation in the tissue is 70 cm/s [32]. The decrement in CV is believed to increase the possibility of reentry occurring, resulting in reduced mechanical responses. As shown in Figure 3(c), LV pressure slightly decreased with a reduction in the CV in SR with a slope of 0.7 and 1.8, respectively. Although the stroke volume remained approximately the same for each slope, they shifted to the meaning that there was a slight weakening of the contraction. Our result is in agreement with the findings of Yuniarti and Lim [33]. The role of CV in chaotic conditions can be seen in Figure 5. The average peak of LV pressure with the CV of 69 cm/s was higher than that with the CV of 31 cm/s in tachycardia condition, although this is not clearly shown in fibrillation condition and needs further investigation.

In chaotic conditions, EAT and MAT were shortened with increased CV (Figures 4, 6, and 7). However, EMD in this condition did not show the same pattern. EMD under the CV of 51 cm/s was longer than that under the CV of 31 cm/s (Table 1). The mechanical responses in tachycardia and fibrillation are shown in Figure 5 in the form of LV and systemic artery pressures for 7 sec. In general, LV and SA pressures in tachycardia showed a steady oscillation compared to those in fibrillation conditions for the CVs of 51 cm/s and 60 cm/s. The mechanical responses in tachycardia and fibrillation conditions for the CV of 31 cm/s were reduced due to S1 stimuli at the first 2.5 s. This was the reason the series of average EMD measurements of the CV of 31 cm/s was the least compared to that of other CVs because the measurement began at 2.5 seconds (Figure 1(a)). Although we investigated tachycardia and fibrillation conditions separately in this work, many researchers have described that tachycardia is actually the initiation stage of fibrillation condition [20, 34, 35].

There are some limitations to this study. The simulation duration of the samples we presented in this study was less than 10 seconds. Particularly, tachycardia and fibrillation data of the CV 31 cm/s were reduced to 36% due to S1 stimuli. More data would be valuable to observe the behavior of EMD in chaotic conditions. In addition, the Purkinje network is not implemented in this study to simulate the tachyarrhythmia and ventricular fibrillation conditions. The electrical signal induced by the Purkinje is prone to affect the EMD distribution. Furthermore, in this electromechanical model, the papillary muscle is not considered. The inclusion of the papillary muscle model is suggested to ensure the role of the papillary muscle to the EMD distribution [3].

## 5. Conclusions

In conclusion, this study observed and quantified EMD distribution in chaotic conditions including tachycardia and ventricular fibrillation. EMD in these chaotic conditions was shorter than that in the SR condition, which supports our hypothesis. This simulation study has expanded our understanding of EMD distribution in the 3D ventricle.

## Figures and Tables

**Table 1:** EMD comparison between sinus rhythm slopes 0.7 and 1.8, tachycardia, and ventricular fibrillation conditions in milliseconds.

Conduction velocity	EMD in SR slope 0.7	Avg. EMD in tachycardia	EMD in SR slope 1.8	Avg. EMD in fibrillation
31 cm/s	66.35	58.61	72.25	53.45
51 cm/s	66.41	58.33	72.78	55.07
69 cm/s	66.60	52.50	72.90	50.77

## Data Availability

The methods and results data used to support the findings of this study are included in the article.
